# Non-primary breast malignancies: a single institution’s experience of a diagnostic challenge with important therapeutic consequences—a retrospective study

**DOI:** 10.1186/s12957-016-0915-4

**Published:** 2016-06-23

**Authors:** Florian E. Buisman, Linda van Gelder, Marian B. E. Menke-Pluijmers, Bob H. C. Bisschops, Peter W. Plaisier, Pieter J. Westenend

**Affiliations:** Department of Surgery, Albert Schweitzer Hospital, Dordrecht, The Netherlands; Department of Surgery, Erasmus MC, University Medical Center Rotterdam, PO Box 2040, 3000 CA Rotterdam, The Netherlands; Department of Radiology, Albert Schweitzer Hospital, Dordrecht, The Netherlands; Laboratory of Pathology, Albert Schweitzer Hospital, Dordrecht, The Netherlands

**Keywords:** Breast, Malignancies, Metastases

## Abstract

**Background:**

Breast cancer is a common malignancy, but metastases to the breast of extramammary malignancies are very rare. Treatment and prognosis are different. The aim of the study is to report the incidence of lymphomas and metastases to the breast of extramammary malignancies in our 30-year archive.

**Methods:**

The pathology database of a single institute was reviewed for all breast neoplasms which were coded in our system as a *metastasis* in the period 1985–2014. Metastatic tumors from primary breast carcinoma were excluded.

**Results:**

A total of 47 patients were included (7 men/40 women, mean age 63 years). The majority originated from lymphoma (*n* = 18) and primary melanoma (*n* = 11). Other primary tumor sites included the ovary (*n* = 6), lung (*n* = 6), colon (*n* = 3), kidney (*n* = 1), stomach (*n* = 1), and chorion (*n* = 1). In 24/47 patients (51 %), metastasis was the first sign of the specific malignant disease. In seven patients (15 %) surgery was performed, the diagnosis of metastatic disease was adjusted in four patients (9 %) postoperatively.

**Conclusions:**

It is important to distinguish lymphomas and metastases to the breast from common primary breast carcinoma for proper treatment and prognosis. Therefore, we emphasize the need for a histological or cytopathological diagnosis before any treatment is commenced. The pathologist plays a key role in considering the diagnosis of metastasis if the histological features are unusual for a primary breast carcinoma. The pathologist should therefore be properly informed by the clinical physician although lymphomas and metastases to the breast are the first presentation of malignant disease in half the cases.

## Background

Metastases from extramammary malignant neoplasms are very rare, since they account for less than 2 % of all breast malignancies [1]. In general, symptoms are similar in those of primary mammary carcinoma, including palpable relatively well-circumscribed and freely movable masses within the breast, pain, tenderness, and inflammation. Non primary breast malignancies are generally well defined and rounded, and calcification is unusual. On mammography, these lesions can be mistaken for benign (fibroadenoma) as well as primary breast malignancies [1, 2].

Metastasis from many extramammary neoplasms have been described, such as lymphomas, malignant melanoma, carcinomas of the lung, ovary, prostate, kidney, stomach, small bowel, large bowel, thyroid, cervix, and soft tissues [1, 3–8].

Distinction of metastases to the breast from extramammary neoplasms from primary breast neoplasms is important for determining the best management. In patients with metastatic disease, a systemic and palliative approach is usually more effective. Moreover, correct diagnosis may prevent unnecessary surgery. The aim of this study is to report the incidence of lymphomas and metastases to the breast of extramammary malignancies in our 30-year archive.

## Methods

The pathology database of a single institute was used to identify all breast neoplasms which were coded in our system as a *metastasis* in the period of 30 years (1985–2014). Each case was retrospectively reviewed by an experienced pathologist in order to determine whether or not the malignancy was rightly coded as non primary breast malignancy. We also included secondary metastases from leukemia-lymphomas to the breast. For all patients, histological reports were reviewed to determine the primary site of the tumor. We reviewed the first manifestation of the primary tumor. Patients with metastatic tumors from primary (contralateral) breast carcinoma were excluded. Additional data were obtained from the hospital electronic record system, retrospectively.

## Results

In the study period, approximately 9000 breast cancers were diagnosed in our laboratory. Fifty-nine of these patients with breast neoplasms were coded as a *metastasis*. In total, 12 patients were excluded because of the following reasons: six were coded incorrectly, they were diagnosed with primary breast cancer, three were excluded because of incomplete information, one was diagnosed with a metastatic tumor from primary breast carcinoma, and two were skin tumors involving the breast, respectively, one squamous cell carcinoma and one primary T cell lymphoma. The final study group comprised 47 patients (Table 1). Females were more frequently affected (7 men, 40 women). The mean age was 63 years (range 32–87). The metastases appeared on average after 39 months (range 1–273) after diagnosis of primary extramammary malignancy. In 24 of the 44 patients (51 %), the metastasis was the first sign of the specific malignant disease.

**Table 1:** Patients characteristics

Age, years	Years
Mean	63
Range	32–87
Sex	Number of patients (%)
Men	7 (15)
Women	40 (85)
Time interval diagnosis: primary neoplasm metastasis (incl. lymphomas)	Months
Mean	39
Range	1–273
Known malignant disease (incl. lymphomas)	Number of patients (%)
No history of primary malignant disease	24 (51 %)
History of primary malignant disease	23 (49 %)

The histology of extramammary breast neoplasms is shown in Table 2. The most common primary neoplasm was lymphoma, followed by melanoma. Other primary tumor sites included the ovary, lung, colon, kidney, stomach, and the chorion. In three patients with melanoma (27 % [3/11]), no primary site was found, most probably due to complete regression of a skin melanoma. All 18 mammary lymphomas were B cell type lymphomas (13 primary, 5 secondary).

**Table 2:** Histology of breast metastases

Primary histology	Number of patients (%)	Interval (months): primary-secondary (mean)
Lymphoma	18 (38.3 %)	
Primary	13	
Secondary	5	5–64 (24)
Melanoma	11 (23.4 %)	
No primary site	3	
Primary side detected	8	4–273 (66)
Ovarian carcinoma	6 (12.8 %)	
No history of ovarian carcinoma	1	
History of ovarian carcinoma	5	1–43 (15)
Lung carcinoma	6 (12.8 %)	
No history of lung carcinoma	4	
History of lung carcinoma	2	10–28 (19)
Adenocarcinoma of the colon	3 (6.4 %)	
No history of colon carcinoma	1	
History of colon carcinoma	2	1–5 (3)
Renal cell carcinoma	1 (2.1 %)	
No history of renal cell carcinoma	0	
History of renal cell carcinoma	1	2 (−)
Adenocarcinoma of the stomach	1 (2.1 %)	
No history of stomach carcinoma	1	
History of stomach carcinoma	0	–
Choriocarcinoma	1 (2.1 %)	
No history of choriocarcinoma	1	
History of choriocarcinoma	0	–
Total	47 (100 %)	

The diagnosis in our group of 47 patients was obtained by core biopsy or with fine-needle aspiration (Table 3). In total, core biopsy was performed in 32 patients (68 %), 14 patients (30 %) had a fine-needle aspiration, and one patient (2 %) was operated without preoperative diagnostics; in this case, an intra-operative frozen section was performed. Seven patients (15 %) had breast surgery. All underwent modified radical mastectomy; the postoperative pathology revealed three melanomas, two ovarian carcinomas, one lymphoma, and one adenocarcinoma of the colon. In these patients, the diagnosis was obtained by core biopsy in three; in two of them, the preoperative diagnosis of primary breast cancer was incorrect retrospectively (i.e., primary breast carcinoma). The remaining patient had a correct preoperative diagnosis, and the surgery was performed for palliation, i.e., local control. Three patients had breast surgery after diagnosis obtained by fine-needle aspiration; in one, there was an incorrect preoperative diagnosis (i.e., primary breast carcinoma), and two patients underwent palliative surgical resection. One patient had no preoperative pathological diagnosis; however, the intra-operative frozen section revealed breast carcinoma, and after postoperative analyses, this diagnosis was adjusted with ovarian carcinoma.

**Table 3:** Type of diagnostics and surgery

Type	Number (%)	Surgery (*n*)	Final diagnosis after surgery
Core biopsy	32 (68)	3	- Lymphoma (*n* = 1) - Melanoma (*n* = 1) - Ovarian carcinoma (*n* = 1): palliative resection^a^
Fine needle aspiration	14 (30)	3	- Melanoma (*n* = 2): palliative resection^a^ - Colon carcinoma (*n* = 1)
Frozen section	1 (2)	1	- Ovarian carcinoma (*n* = 1)
Total (*n*)	47 (100)	7	

^a^Correct preoperative diagnosis

## Discussion

Breast metastases of extramammary neoplasms are extremely rare. In our laboratory, we found a total of 47 patients with metastases to the breast from non mammary primary in a 30-year period, representing an incidence of approximately 0.5 %. In our study, lymphomas and melanomas were the most prevalent primary neoplasms (38 and 23 %, respectively), which is in accordance with previous reported studies [1, 2, 4, 9]. Lee et al. reported gastric cancers to be the most common metastases to the breast. However, gastric carcinoma is the most common form of cancer in Korea and the incidence of melanoma is much lower [10].

In our study, patients with breast metastases presented with a mean of 39 months after diagnoses of primary cancer. Patients with primary melanomas presented with the largest time interval between primary presentation and breast metastases with a mean of 66 months, probably reflecting the well-known natural history of melanoma with a subgroup showing late recurrence. Lee et al. reported a mean time interval of 14 months, but this difference in interval might be explained by the larger amount of melanomas included in our study (23 vs 6 %) [10].

In this study, in four patients (9 %), the diagnosis had to be corrected postoperatively. This percentage seems poor; however, it should be considered that diagnosis of extramammary metastasis to the breast is difficult not only because clinical presentation may be similar to primary breast malignancies but also because it may be the first presentation of unknown metastatic disease. In addition, the diagnosis is hard to make on cytology alone. Obviously, proper clinical information may be helpful and should be provided to the pathologist at all times.

Histological diagnosis is important to distinguish metastases to the breast from common primary breast carcinoma for effective treatment and direct attention to the extramammary primary, and in case of metastases to the breast prevent unnecessary surgery. Lee et al. reviewed histological and immunohistochemical features that are useful in the diagnosis of metastases to the breast. They discovered that about a third of the lesions do not show specific histological features. Elastosis and carcinoma in situ are common histological characteristics in primary mammary cancers. Different types of carcinoma are suggestive for specific origins, such as clear cell carcinoma for renal origin, small cell carcinoma of pulmonary origin, and papillary carcinoma of ovarian serous papillary carcinoma. For malignant melanomas, the appearance of cytoplasmic pigment, intranuclear inclusions, and spindle cells may be useful clues [11].

Immunohistochemical panels can be of great value when trying to distinguish mammary carcinoma from common metastases to the breast. A mammary origin can be supported by the expression of estrogen receptor, progesterone receptor, her2neu, and GCDFP-15. Recently, GATA-3 has been shown to be a sensitive marker for breast cancer [12, 13]. However, these markers are not entirely specific, e.g., estrogen receptor can be expressed in ovarian cancer and GATA-3 is also a sensitive marker for urothelial cancer. Sensitive markers for the metastases that we found in our series are summarized in Table 4 [14, 15]. These markers should always be used in a panel of antibodies since no single marker is completely sensitive or specific. These markers are readily available on core biopsies but can also be applied to cytological specimens.

**Table 4:** Sensitive markers for specific metastases, these markers are negative in breast cancer

Primary origin	Marker
Lung adenocarcinoma	TTF-1
Lung small cell carcinoma	TTF-1, CD56
Melanoma	HMB45, Melan-A
Ovarian cancer	Pax-8, WT1, Ca 125
Colorectal carcinoma	CDX2, CK20
Malignant B cell lymphoma	CD20, CD79a
Renal cell carcinoma	Pax-5, RCC
Choriocarcinoma	HCG-beta

Several authors emphasized the usages of fine-needle cytology [16–19]. Fulciniti et al. emphasized the usage of cytopathological criteria in combination with immunocytochemistry when considering the diagnosis of metastases to the breast [16]. As stated before, diagnosis of metastases to the breast of non primary breast malignancies should be performed by a combination of clinical presentation and evaluation of the pathology and specific antibodies.

## Conclusions

It is concluded that although metastases to the breast are rare, it should be considered in the differential diagnosis of breast cancer if the morphology is unusual for primary breast malignancies and if there is a history of a prior (treated) malignancy, in order to avoid unnecessary surgery.

## Abbreviations

Ca 125, cancer antigen 125; CD56, cluster of differentiation 56; CD79a, cluster of differentiation 79a; CDX2, cluster of differentiation X2; CK20, cytokeratin 20; GCDFP-15, gross cystic disease fluid protein-15; HCG-beta, human chorionic gonadotropin-beta; HMB45, human melanoma black 45; Melan-A, melanoma antigen; Pax-5, paired-box gene 5; Pax-8, paired-box gene 8; RCC, renal cell carcinoma marker; TTF-1, thyroid transcription factor 1; WT1, Wilms tumor 1
